# Intravenous Ceftriaxone Versus Multiple Dosing Regimes of Intravenous Anti-Staphylococcal Antibiotics for Methicillin-Susceptible *Staphylococcus aureus* (MSSA): A Systematic Review

**DOI:** 10.3390/antibiotics9020039

**Published:** 2020-01-21

**Authors:** Musaiwale M. Kamfose, Francis G. Muriithi, Thomas Knight, Daniel Lasserson, Gail Hayward

**Affiliations:** 1 Department of Infection Control and Microbiology, John Radcliffe Hospital, Headley Way, Oxford OX3 7JS, UK; 2 Obstetrics and Gynaecology, Queen’s Medical Centre, Nottingham University Hospitals NHS Trust, Derby Rd, Nottingham NG7 2UH, UK; francis.muriithi@nhs.net; 3 Institute of Applied Health Research, University of Birmingham, Birmingham B15 2TT, UK; thomasknight@nhs.net (T.K.); Daniel.Lasserson@nhs.net (D.L.); 4 Nuffield Department of Primary Care Health Sciences, Radcliffe Primary Care Building, Radcliffe Observatory Quarter, Woodstock Road, Oxford OX2 6GG, UK; gail.hayward@phc.ox.ac.uk

**Keywords:** intravenous ceftriaxone, anti-staphlococcal antibiotics, mssa, multiple dosing

## Abstract

Background: Methicillin-susceptible *Staphylococcus aureus* (MSSA) is a common pathogen associated with a range of clinically important infections. MSSA can cause deep-seated infections requiring prolonged courses of intravenous antibiotic therapy to achieve effective resolution. The move toward ambulatory or outpatient delivery of parenteral antibiotics has led to an increase in the use of ceftriaxone as a pragmatic first choice given its advantageous single daily dosing schedule. Objective: To compare the efficacy of once daily ceftriaxone in the treatment of infections due to confirmed or suspected MSSA to multiple dosing regimes of anti-staphylococcal antibiotics. Methods: We searched the Cochrane Central Register of Controlled Trials (CENTRAL), Database of Abstracts of Reviews of Effects (DARE), Global Health, PubMed, EMBASE and CINAHL for randomised controlled trials as well as prospective and retrospective cohort studies that compared ceftriaxone to any multiple dosing regime of anti-staphylococcal antibiotics. Outcome measures were the proportion of patients with a resolution of infection based on time after initiation of therapy, adverse reactions, recurrence and duration of hospital admission. Results: We included two randomized controlled trials, one prospective observational study and three retrospective cohort studies (643 participants; 246 children, 397 adults). There was no difference in time to resolution of symptoms. The number of adverse reactions, recurrence of bacteraemia and duration of hospital stay were not significantly different between ceftriaxone and other anti-staphylococcal antibiotics. Conclusions: Based on a small number of low-quality studies, ceftriaxone is as effective as multiple dosing regimes for the treatment of infections due MSSA. An appropriately powered randomized trial is required to demonstrate equivalence and cost effectiveness.

## 1. Background

*Staphylococcus aureus* is a common pathogen implicated in soft tissue infection, infection of bones and joints and endocarditis. The severity of infection ranges from localised cellulitis, minor boils or skin abscesses to life-threatening systemic infection associated with bacteraemia. Intravenous beta-lactam antibiotics such as anti-staphylococcal penicillins are recommended as first line treatment for MSSA infection [1]. The management of *S. aureus* infection no longer necessitates inpatient admission for the duration of treatment. Services delivering intravenous antibiotics in the ambulatory care or home setting are considered credible alternatives to bed based care in patients who do not require organ system support or intensive physiological monitoring [2]. This approach has the potential to improve patient experience, reduce harm associated with inpatient admission and reduce costs of care [3]. In order to realise these advantages, changes to standard treatment regimens have been adopted which minimise the frequency of drug administration.

While anti-staphylococcal penicillin regimens are well established and efficacious treatments in the management of MSSA infection, the requirement for multiple daily dosing schedules to achieve adequate therapeutic concentrations is a significant impediment to its use in the home or ambulatory setting. Ceftriaxone is a third-generation broad-spectrum cephalosporin with in vitro activity against MSSA [4]. Ceftriaxone is extensively protein bound, resulting in a long elimination half-life that allows one to only take one daily dosing [3]. Ceftriaxone has become an attractive alternative to treat confirmed or suspected MSSA and accounts for 18% of all outpatient parenteral antibiotic prescriptions in Europe [5]. Ceftriaxone administered at a dose of 2 g IV q24 h is recommended by the Infectious Disease Society of America as a potential first line treatment for native vertebral osteomyelitis and prosthetic joint infection secondary to MSSA [6]. Ceftriaxone can also be used to treat skin or soft tissue infections in patients who are deemed to require parenteral antibiotics but are otherwise suitable for ambulatory or outpatient management [7].

The pharmacokinetic properties of ceftriaxone offer significant practical advantages over other first line antibiotic regimes targeted at MSSA, but questions remain regarding the efficacy and safety of this approach. Theoretical concerns have been raised regarding impaired bactericidal activity of cephalosporins secondary to altered pharmacodynamics caused by an inoculum effect [8]. The use of ceftriaxone in the initial management of MSSA bacteraemia is controversial given the potential severity of the condition and the limited available clinical evidence to support its use [9]. A multi-centre prospective clinical study of 77 patients treated with the narrow-spectrum anti-staphylococcal cephalosporin, cefazolin, for MSSA bacteraemia, reported an association between the cefazolin-induced inoculum effect and increased 30-day mortality [10]. Whether these concerns are clinically significant or generalisable to ceftriaxone is unclear. In addition to concerns regarding efficacy, the use of ceftriaxone in preference to antibiotics with a narrower spectrum of activity may also contribute to antimicrobial resistance and increase the risk of *Clostridium difficile* infection [11].

We performed a systematic review to establish the efficacy of a single daily dose of ceftriaxone for the treatment of suspected or confirmed MSSA infection in comparison with multiple dose regime anti-staphylococcal antibiotic regimens.

## 2. Methods

### 2.1. Inclusion Criteria

We included studies of children and adults with suspected methicillin-susceptible *S. aureus*, defined by clinical diagnosis, where ceftriaxone was compared to other multiple-dose antibiotic regimes. We defined multiple-dose regimens as any antibiotic schedule requiring more than one dose within a 24 h period. The specific type of antibiotic used in the comparator group was not pre-specified. Studies were included that utilised ceftriaxone across a range of clinical infections. Microbiological confirmation of MSSA was not a prerequisite for inclusion. While this approach led to a more heterogenous study population it was felt to more accurately reflect the use of ceftriaxone in real world practice where antibiotic choice is frequently undertaken in advance of, or in the absence of identification of a specific pathogen. We excluded trials where a definitive diagnosis of methicillin-resistant *S. aureus* (MRSA) was made, adjunctive therapy was used in one arm and studies that lacked a comparator or that were conducted in vitro.

We included randomised controlled trials, as well as prospective and retrospective cohort studies ascertaining the efficacy of ceftriaxone compared to any multiple anti-staphylococcal antibiotic. We included trials reporting combined interventions if they allowed a direct comparison between ceftriaxone and any standard anti-staphylococcal antibiotics.

### 2.2. Outcome Measures

#### 2.2.1. Primary Outcome

The proportion of participants with resolution or improvement of symptoms after initiation of appropriate therapy.

#### 2.2.2. Secondary Outcomes

Adverse reaction to antibiotics used in the treatment of infections due to *S. aureus* bacteraemia, recurrence of bacteraemia within 60 days of discontinuing therapy and the duration of any hospital admission.

### 2.3. Search Methods for Identification of Studies

We searched the Cochrane Register of Controlled Trials (CENTRAL 2019, Issue 7), which also includes the Infectious Diseases (ID) group’s Specialised Register, the Database of Abstracts of Reviews of Effects (DARE) 2019, Issue 7, on the Cochrane Library (Wiley), Global Health (1973 to July 2019), PubMed/MEDLINE (OvidSP) (1982 to July 2019), EMBASE (OvidSP) (1974 to July 2019) and CINAHL (1996 to July 2019). Details of the search strategies are in Supplementary Files 1–5. We conducted follow up searches relevant to the study using the citation lists of papers identified by the above strategies. Title, abstract screening and data extraction were performed by one reviewer (MK) and cross checked by a second reviewer (FM), with disagreements resolved by discussion with a third author (GH).

### 2.4. Quality Assessment of the Included Studies

Two authors independently assessed the methodological quality of the selected articles. We used the Cochrane “Risk of bias” tool for randomised controlled trials (RCTs) and the ROBINS-I tool for assessing the quality of non-randomised studies.

## 3. Results

**Figure 1:**
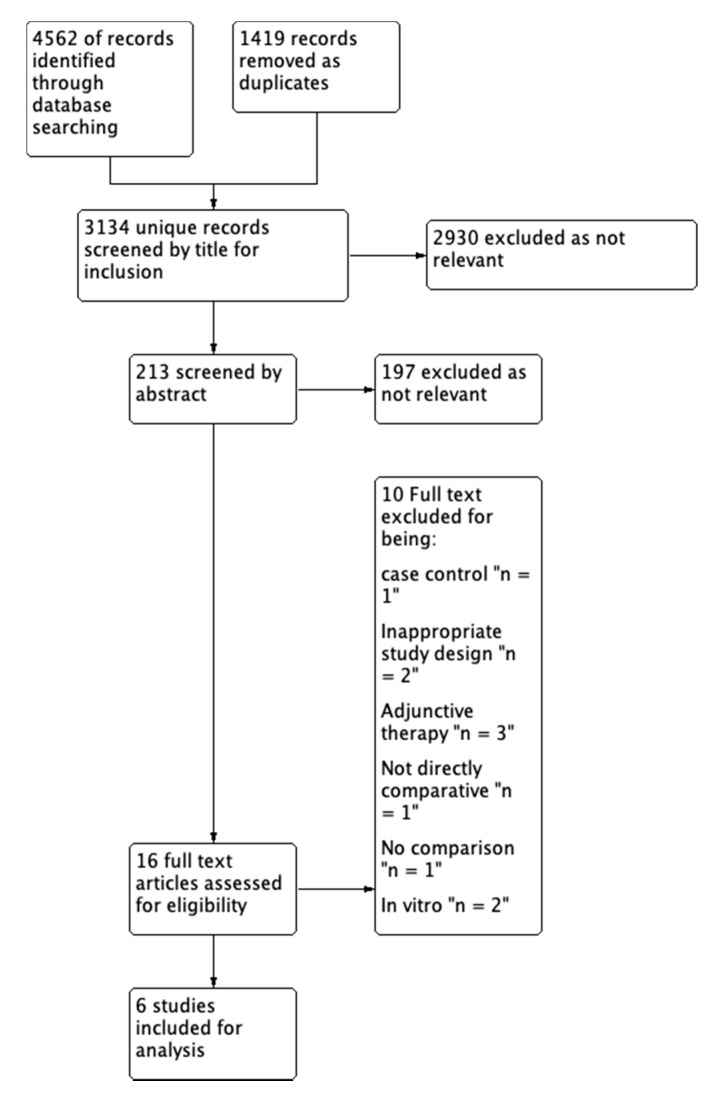
PRISMA flow chart.

We identified 4562 studies (Figure 1). After removal of duplicates, 3143 studies remained, 2930 articles were excluded after title screening and a further 197 articles excluded after abstract screening. We obtained full-text copies of 16 articles and excluded 10. Of these, one study had clinical outcome measures different to other studies under review [12]. Three studies used ceftriaxone as an adjunctive therapy for the treatment of MSSA or did not describe MSSA infections [13,14,15]. Two studies were in vitro models [4,16], one [17] lacked a comparator and one study did not use ceftriaxone [18]. Two studies [19,20] assessed a population that presented with pneumonia not demonstrated to be due to MSSA.

### 3.1. Included Studies

We included two randomised controlled trials (RCTs) [21,22], one observational prospective study and three retrospective cohort studies [23,24,25,26]. Two studies were conducted in Australia [22,26] and the rest [21,23,24,25] in the USA. In total the studies involved 673 participants (246 children and 427 adults) investigated or diagnosed with MSSA. See Table 1 for characteristics of included studies.

All included studies assessed the number of participants who had either partial or complete resolution of signs and symptoms of infection due to suspected or confirmed MSSA infection. Four studies [22,23,25,26] assessed adverse events and the duration of treatment. Other outcomes assessed were recurrence of infection necessitating readmission [25,26] and cost of care [22,24,25,26].

Both randomised controlled trials [21,22] were funded by pharmaceutical companies (Pfizer Pharmaceutical Company and Roche Products PVT Limited, respectively), and at the time of publication Roche owned the ceftriaxone patent. Two studies were funded by charitable organisations and government institutions [24,26] and two studies reported no funding [23,25].

### 3.2. Risk of Bias in Included Studies (RCTs)

**Figure 2:**
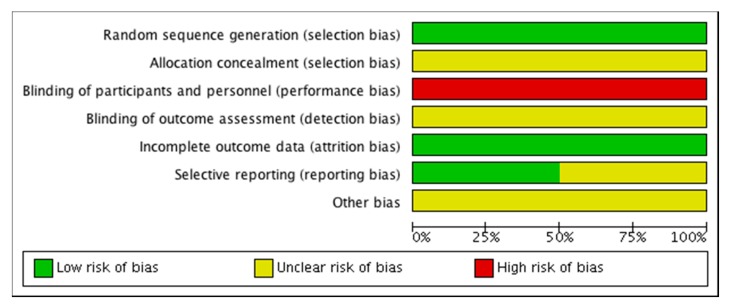
Risk of bias in the randomised controlled trials.

One randomized controlled trial [21] used computer generated randomisation, and the other did not specify the method [22], see Table 2. The proportions of low, moderate, critical and serious risk of bias in each domain are illustrated in Figure 2.

We used the ROBINS-I tool [27] to assess the quality of non-randomised studies (Table 3). Key limitations identified included study design (lack of parallel group randomised trials); use of cohorts in different clinical settings and age groups (i.e., hospital in the home patients were older and hospitalised group younger); and potential for confounding of effects due to previous antibiotics prior to admission. The lack of an objective outcome measure to determine response, combined with the assessor’s knowledge of treatment allocation was also identified as a consistent source of potential bias. All studies assessed demonstrated significance in at least one domain.

### 3.3. Effects of Interventions

#### 3.3.1. The Proportion of Participants with Resolution or Improvement of Symptoms

##### Randomised Controlled Trials

Both RCTs (179 participants—132 children and 47 adults) reported data on the proportion of resolution or improvement of symptoms. One study [21] compared ampicillin/sulbactam vs. ceftriaxone while the other [22] compared flucloxacillin vs. ceftriaxone. The follow-up period for outcome assessment was six months and 48 h–72 h, respectively [21,22]. One study [21] defined a resolution as the elimination of the initial pathogen during therapy and resolution of clinical signs of infection at the end of hospitalisation. The other [22] defined a resolution or improvement as complete resolution of all signs and symptoms of *S. aureus* bacteraemia after completion of parenteral therapy at either 48 or 72 h (Table 4). There were no statistically significant differences in improvement or resolution between the control and intervention groups. Given the variation in outcome assessment and antibiotics, it was not possible to combine data for these two studies.

##### Cohort Studies

(1) Ceftriaxone compared to beta lactams:

Two studies [24,26] compared ceftriaxone to flucloxacillin and oxacillin, respectively. Resolution was defined as resolution of signs and symptoms of infection, improvement in inflammatory markers and no requirement for readmission at follow-up by one study [24] and as the absence of treatment failure (hospital readmission because of inadequate improvement at home determined by a clinician) by the other [26]. Outcome measures were assessed between three and six months in a follow-up visit and at six months after completion of intravenous antibiotic [24]. Neither study found a significant difference in the proportion of patients with a resolution of symptoms between ceftriaxone and the beta-lactam comparator.

(2) Ceftriaxone compared to other anti-staphylococcal antibiotics:

Two studies compared ceftriaxone versus cefazolin, nafcillin or vancomycin [16] and ceftriaxone versus cefazolin [23,25]. Resolution of staphylococcal infection was defined as a favourable clinical outcome measured by a physician’s expectations of outpatient parenteral antimicrobial therapy (OPAT) for MSSA infection [25] or as resolution of signs and symptoms related to bacteraemia, with no occurrence within six months of completion of antibiotic therapy [23]. Outcome measures were assessed at three months and 12 months in one study [25] and six months in the other [23]. In both studies, the proportion of patients experiencing resolution was not significantly different in the ceftriaxone cohort compared to other anti-staphylococcal antibiotics (Table 5). 

### 3.4. Secondary Outcomes

#### 3.4.1. Duration of Hospital Admission

Table 4 describes the duration of hospital stay in our two included RCTs [21,22]. No significant differences were found between ceftriaxone and multiple dosing regimes. Three cohort studies [23,25,26], found no significant difference in the duration of hospital admission. The remaining cohort study [24] did not report on this outcome (see Table 5).

#### 3.4.2. Recurrence of the Bacteraemia or Symptoms within 60 Days of Discontinuing Therapy

The three cohort studies which reported this outcome found no differences between ceftriaxone and other antibiotic groups.

#### 3.4.3. Adverse Reaction to Antibiotics

No significant differences were found in the adverse reactions in the two RCTs. One study [21] reported mild elevation in blood urea nitrogen and diarrhoea. The other study [22] reported vaginal candidiasis, nausea and diarrhoea in the ceftriaxone group, and nausea, diarrhoea and abdominal pain in the flucloxacillin group (see Table 4). In the three cohort studies reporting this outcome, we found no differences in adverse events between ceftriaxone and the comparator antibiotic (see Table 5).

## 4. Discussion

### 4.1. Summary of Main Findings

We found two RCTs and four cohort studies comparing ceftriaxone to multiple dosing regimes for MSSA infections. Although the definition of resolution differed significantly between studies, all included studies found no difference in time to resolution of infection, duration of hospital admission, recurrence of bacteraemia or adverse events when comparing ceftriaxone to multiple dosing regimes. However, these results are based on a small number of low-quality studies, including small numbers of participants and using mainly observational designs. The association between ceftriaxone use and outpatient or ambulatory management creates a selection bias towards less severe illness, which is likely to favour the intervention.

### 4.2. Strengths and Limitations

This review performed a comprehensive search of several databases with independent screening, selection and assessment of eligible articles. The clinical studies included were heterogenous with significant differences in the primary focus of infection under investigation, the antibiotic regimens used as comparators and the methodologies and assessment periods used in outcome measurement. A conscious decision was made to include studies based on the presence of a primary focus of infection compatible with MSSA infection rather than microbiologically confirmed MSSA infection. We felt this pragmatic approach best represented the current use of ceftriaxone in clinical practice, where MSSA infection is often suspected on clinical grounds but never definitively confirmed. This approach inevitably resulted in clinical heterogeneity between the included studies.

### 4.3. Comparison with other Literature

We are not aware of any other systematic review on this topic. Two retrospective cohort studies not eligible for inclusion in this review demonstrated no clear difference between ceftriaxone and alternative antibiotics for MSSA [12,18]. A recent meta-analysis comparing ceftriaxone with ceftaroline or ceftobiprole identified five RCTs and a total of 77 patients with community-acquired pneumonia secondary to MSSA. Patients treated with ceftriaxone demonstrated higher rates of treatment failure [28]. None of our included studies contained patients with MSSA pneumonia or comparator groups treated with 5th generation cephalosporins. MSSA pneumonia is relatively uncommon and associated with a high case fatality [29]. The findings of our study are more relevant to the treatment of suspected MSSA infections in the ambulatory or outpatient setting.

### 4.4. Implications for Practice and Research

Based on the limited evidence available, this review suggests that the practice of using ceftriaxone for the treatment of *S. aureus* infection is safe and effective when compared to alternative antibiotic therapies. However, large randomised controlled trials are needed to evaluate the effectiveness of ceftriaxone compared to multiple doses of anti-staphylococcal antibiotics in the treatment of infections due to MSSA. Such trials should include measures of symptom improvement and resolution and rates of representation to healthcare, and should additionally evaluate the cost effectiveness of the two approaches in settings where ambulatory care is possible.

## 5. Conclusions

It is imperative that antibiotic selection in the management of MSSA infection is predicated on strong clinical evidence of efficacy alongside practical considerations surrounding the ideal care setting. Based on a small number of low-quality studies, ceftriaxone is as effective as multiple dosing regimes for the treatment of infections due to MSSA. An appropriately powered randomized trial is required to demonstrate equivalence and cost effectiveness.

## Figures and Tables

**Table 1:** Charicharistics of included studies.

Study	Setting	Study Type	Age of Patients	*n*	Intervention/Duration	Comparator/Duration	Outcome
Kulhanjian et al. 1989 [21]	Oakland, CA	RCT	2 months–18 years	132	Ceftriaxone 50–70 mg/kg/24 h	Ampicillin 15–30 mg/kg/6 h	Resolution of clinical signs and symptoms of infection at the end of hospitalisation. Absences of recurrence of symptoms on follow up. Elimination of initial pathogen during therapy. Delayed response to treatment.
Vinen et al. 1996 [22]	Royal North Shore Hospital, Australia	RCT	19–61 years	58	Ceftriaxone 1 gram/24 h	Flucloxacillin 1 gram/6 h	Complete resolution of all signs and symptoms of infection. Improvement but without complete resolution of signs and symptoms. Eradication of causative pathogen.
Ibrahim et al. 2016 [26]	Melbourne Australia	Retrospective cohort	3 months–18 years	114	Ceftriaxone 50 mg/kg/24 h	Flucloxacillin 50 mg/kg/6 h	Treatment failure. Defined as admission from hospital in the home HITH back to hospital because of inadequate improvement at home as determined by the treating clinician Change of antibiotic because of poor clinical improvement. Septic complication or recurrence during use or within 48 h of I.V. Adverse events. Death within 28 days Length of hospital stay.
Winans et al. 2013 [25]	Houston Texas	Retrospective cohort	18–78 years	122	Ceftriaxone 1 g & 2 g/24 h	Cefazolin 2 g/8 h	Favourable clinical outcome-no signs and symptoms of infection. Complications as a result of infection. readmission to hospital. Adverse events-nausea/vomiting or diarrhoea, Anaemia, worsening infection.
Wieland et al. 2012 [24]	St Louis Missouri	Retrospective cohort	>18 years	124	Ceftriaxone 2 g/24 h	Oxacillin 4 g/6 h	Duration of I.V antibiotic. Resolution of signs and symptoms of infection, Improvement in inflammatory markers. Complications due to toxicity. Repeat surgery or readmission.
Patel et al. 2014 [23]	Chicago	Retrospective cohort	25–87 years	93	Ceftriaxone 2 g/24 h	Nafcillin/Cefazolin/Vancomycin 41 ± 38 days	Clearance of MSSA bacteraemia. Cure of source and/or complication of MSSA bacteraemia. No recurrence within 6 months of completion of I.V therapy. Resolution of signs and symptoms related to bacteraemia.

**Table 2:** Risk of bias in the included studies (randomised controlled trials (RCTs)).

Study	Allocation Bias	Blinding	Incomplete Outcome	Selective Reporting	Other Potential Sources
Kulhanjian et al. 1989 [21]	Computer-generated in a 2:1 fashion. Low risk of bias	Insufficient information on blinding High risk bias. Did not report on the blinding of outcome Unclear risk of bias.	Insufficient information Unclear risk bias	No evidence for selective reporting Low risk of bias	Funded by pharmaceutical company No declarations of conflict of interest made.
Vinen et al. 1996 [22]	Details of randomization not provided Low risk of bias because of the nature of the study but unclear risk due to the study design.	Insufficient information on blinding of participants and researchers High risk of bias	High dropout rate. Unclear risk of bias	No evidence for selective reporting Low risk of bias.	Funded by pharmaceutical company No declarations of conflict of interest were made The lack of blinding in the study posed an issue for allocation.

**Table 3:** Risk of bias non-randomised studies.

Eligible Studies	Risk of Bias Domains—ROBINS-I							
	Bias Due to Confounding	Bias in Selection of Participants into the Study	Bias in Classification of Interventions	Bias Due to Deviations from Intended Interventions	Bias Due to Missing Data	Bias in Measurement of Outcome	Bias in the Selection of the Reported Result	Overall Risk of Bias
Ibrahim et al. (2016) [26]	Critical	Serious	Serious	Serious	No Information	Serious	Serious	Critical
Winans et al. (2013) [25]	Moderate	Low	Moderate	Low	No Information	Serious	Serious	Serious
Wieland et al. (2012) [24]	Low	Low	Moderate	Serious	Moderate	Moderate	Moderate	Serious
Patel et al. (2014) [23]	Serious	Serious	Serious	Serious	No Information	Moderate	Moderate	Serious

**Table 4:** Outcomes (randomised studies).

Study	Duration of Hospital Admission	Recurrence within 60 Days of Discontinuing Therapy	Adverse Reaction to Antibiotics
Vinen et al. 1996 [22]	Ceftriaxone mean 9.11 ± 5.49 days flucloxacillin mean 9.87 ± 6.68 days	Ceftriaxone 4.1% (1/24) Flucloxacillin 26% (6/23)	Ceftriaxone 12.5% (3/24) Flucloxacillin 26% (6/23)
Kulhanjian et al. 1989 [21]	Ceftriaxone 5 days Ampicillin/sulbactam 3.5 days (standard deviation not reported)	Ceftriaxone 7.3% (3/41) Ampicillin group 1.2% (1/84)	Ceftriaxone 7.3% (3/41) ampicillin/sulbactam 1.2% (1/84)

**Table 5:** Outcomes (cohort studies).

Study	Duration of Hospital Admission	Recurrence of the Bacteraemia or Symptoms within 60 Days	Adverse Reaction
Patel, McKissic et al. 2014 [23]	Ceftriaxone mean 28 days (SD 44); Nafcillin/cefazolin/vancomycin group mean 36 days (SD 43) (*p* = 0.41).	Ceftriaxone 11.9% (5/42) standard antibiotic 11.7 (6/51)	Ceftriaxone 7.1% (3/42) Standard antibiotic 1.9% (1/51)
Ibrahim et al. 2016 [26]	Ceftriaxone mean 2.7 days (range 1–10); Flucloxacillin 2.7 days (range 1–8)	Ceftriaxone 4.9% (2/41) Flucloxacillin 2.9% (3/103)	Not reported
Winans et al. 2013 [25]	Ceftriaxone median 12 days (range 4–26) Cefazolin median 9 days (range 3–27)	Ceftriaxone 6.8% (3/44) Cefazolin 9.0% (7/78)	Ceftriaxone 2.27% (1/44) Cefazolin 5.1% (4/78)
Wieland et al. 2012 [24]	Not reported	Not reported	Ceftriaxone 2.7% (2/74) Oxacillin 5% (2/40)

## Supplementary Materials

The following are available online at https://www.mdpi.com/2079-6382/9/2/39/s1, Supplementary File 1: PubMed/MEDLINE (OvidSP) (1982 to July 2019). Supplementary File 2: EMBASE (OvidSP) (1974 to July 2019). Supplementary File 3: Cochrane Central Register of Controlled Trials (CENTRAL 2019, Issue 7). Supplementary File 4: Global Health (1973 to July 2019). Supplementary File 5: CINAHL (1996 to July 2019).

## References

[1] McDanel J.S., Perencevich E.N., Diekema D.J., Herwaldt L.A., Smith T.C., Chrischilles E.A., Dawson J.D., Jiang L., Goto M., Schweizer M.L. (2015). Comparative effectiveness of beta-lactams versus vancomycin for treatment of methicillin-susceptible Staphylococcus aureus bloodstream infections among 122 hospitals. Clin. Infect. Dis..

[2] Seaton R.A., Bell E., Gourlay Y., Semple L. (2005). Nurse-led management of uncomplicated cellulitis in the community: Evaluation of a protocol incorporating intravenous ceftriaxone. J. Antimicrob. Chemother..

[3] Caplan G.A., Sulaiman N.S., Mangin D.A., Aimonino Ricauda N., Wilson A.D., Barclay L. (2012). A meta-analysis of “hospital in the home”. Med. J. Aust..

[4] Phe K., Dao D., Palmer H.R., Tama V.H. (2015). In vitro ceftriaxone susceptibility in methicillin-susceptible staphylococcus aureus. Antimicrob. Agents Chemother..

[5] Coenen S., Muller A., Adriaenssens N., Vankerckhoven V., Hendrickx E., Goossens H. (2009). European Surveillance of Antimicrobial Consumption (ESAC): Outpatient parenteral antibiotic treatment in Europe. J. Antimicrob. Chemother..

[6] Berbari E.F., Kanj S.S., Kowalski T.J., Darouiche R.O., Widmer A.F., Schmitt S.K., Hendershot E.F., Holtom P.D., Huddleston P.M., Petermann G.W. (2015). 2015 Infectious Diseases Society of America (IDSA) Clinical Practice Guidelines for the Diagnosis and Treatment of Native Vertebral Osteomyelitis in Adults. Clin. Infect. Dis..

[7] Duncan C.J., Barr D.A., Seaton R.A. (2012). Outpatient parenteral antimicrobial therapy with ceftriaxone, a review. Int. J. Clin. Pharm..

[8] Lenhard J.R., Bulman Z.P. (2019). Inoculum effect of beta-lactam antibiotics. J. Antimicrob. Chemother..

[9] Lother S.A., Press N. (2017). Once-Daily Treatments for Methicillin-Susceptible Staphylococcus aureus Bacteremia: Are They Good Enough?. Curr. Infect. Dis. Rep..

[10] Miller W.R., Seas C., Carvajal L.P., Diaz L., Echeverri A.M., Ferro C., Rios R., Porras P., Luna C., Gotuzzo E. (2018). The Cefazolin Inoculum Effect Is Associated with Increased Mortality in Methicillin-Susceptible Staphylococcus aureus Bacteremia. Open Forum Infect. Dis..

[11] Llor C., Bjerrum L. (2014). Antimicrobial resistance: Risk associated with antibiotic overuse and initiatives to reduce the problem. Ther. Adv. Drug Saf..

[12] Wynn M., Dalovisio J.R., Tice A.D., Jiang X. (2005). Evaluation of the efficacy and safety of outpatient parenteral antimicrobial therapy for infections with methicillin-sensitive Staphylococcus aureus. South. Med. J..

[13] Badaro R., Molinar F., Seas C., Stamboulian D., Mendonca J., Massud J., Nascimento L.O. (2002). A multicenter comparative study of cefepime versus broad-spectrum antibacterial therapy in moderate and severe bacterial infections. Braz. J. Infect. Dis..

[14] Frank E., Liu J., Kinasewitz G., Moran G.J., Oross M.P., Olson W.H., Reichl V., Freitag S., Bahal N., Wiesinger B.A. (2002). A multicenter, open-label, randomized comparison of levofloxacin and azithromycin plus ceftriaxone in hospitalized adults with moderate to severe community-acquired pneumonia. Clin. Ther..

[15] Nicholson S.C., Welte T., File T.M., Strauss R.S., Michiels B., Kaul P., Balis D., Arbit D., Amsler K., Noel G.J. (2012). A randomised, double-blind trial comparing ceftobiprole medocaril with ceftriaxone with or without linezolid for the treatment of patients with community-acquired pneumonia requiring hospitalisation. Int. J. Antimicrob. Agents.

[16] MacVane S.H., So W., Nicolau D.P., Kuti J.L. (2014). In Vitro activity of human-simulated epithelial lining fluid exposures of ceftaroline, ceftriaxone, and vancomycin against methicillin-susceptible and -resistant Staphylococcus aureus. Antimicrob. Agents Chemother..

[17] Frenkel L.D. (1998). Once-daily administration of ceftriaxone for the treatment of selected serious bacterial infections in children. Pediatrics.

[18] Paul M., Zemer-Wassercug N., Talker O., Lishtzinsky Y., Lev B., Samra Z., Leibovici L., Bishara J. (2011). Are all beta-lactams similarly effective in the treatment of methicillin-sensitive Staphylococcus aureus bacteraemia?. Clin. Microbiol. Infect..

[19] Pertel P.E., Bernardo P., Fogarty C., Matthews P., Northland R., Benvenuto M., Thorne G.M., Luperchio S.A., Arbeit R.D., Alder J. (2008). Effects of prior effective therapy on the efficacy of daptomycin and ceftriaxone for the treatment of community-acquired pneumonia. Clin. Infect. Dis..

[20] Cannavino C.R., Nemeth A., Korczowski B., Bradley J.S., O’Neal T., Jandourek A., Friedland H.D., Kaplan S.L. (2016). A Randomized, Prospective Study of Pediatric Patients with Community-acquired Pneumonia Treated with Ceftaroline Versus Ceftriaxone. Pediatr. Infect. Dis. J..

[21] Kulhanjian J.U., Dunphy M.G., Hamstra S.C., Levernier K.A., Rankin M.I., Petru A., Azimi P.A. (1989). Randomized comparative study of ampicillin/sulbactam vs. ceftriaxone for treatment of soft tissue and skeletal infections in children. Pediatr. Infect. Dis. J..

[22] Vinen J., Hudson B., Chan B., Fernandes C. (1996). A randomised comparative study of once-daily ceftriaxone and 6-hourly flucloxacillin in the treatment of moderate to severe cellulitis. Clinical efficacy, safety and pharmacoeconomic implications. Clin. Drug Investig..

[23] Patel U.C., McKissic E.L., Kasper D., Lentino J.R., Pachucki C.T., Lee T., Lopansri B.K. (2014). Outcomes of ceftriaxone use compared to standard of therapy in methicillin susceptible staphylococcal aureus (MSSA) bloodstream infections. Int. J. Clin. Pharm..

[24] Wieland B.W., Marcantoni J.R., Bommarito K.M., Warren D.K., Marschall J. (2012). A retrospective comparison of ceftriaxone versus oxacillin for osteoarticular infections due to methicillin-susceptible staphylococcus aureus. Clin. Infect. Dis..

[25] Winans S.A., Luce A.M., Hasbun R. (2013). Outpatient parenteral antimicrobial therapy for the treatment of methicillin-susceptible Staphylococcus aureus: A comparison of cefazolin and ceftriaxone. Infection.

[26] Ibrahim L.F., Hopper S.M., Babl F.E., Bryant P.A. (2016). Who can have parenteral antibiotics at home? A prospective observational study in children with moderate/severe cellulitis. Pediatr. Infect. Dis. J..

[27] Sterne J.A., Hernán M.A., Reeves B.C., Savović J., Berkman N.D., Viswanathan M., Henry D., Altman D.G., Ansari M.T., Boutron I. (2016). ROBINS-I: A tool for assessing risk of bias in non-randomised studies of interventions. BMJ.

[28] Eljaaly K., Wali H., Basilim A., Alharbi A., Asfour H.Z. (2019). Clinical cure with ceftriaxone versus ceftaroline or ceftobiprole in the treatment of staphylococcal pneumonia: A systematic review and meta-analysis. Int. J. Antimicrob. Agents.

[29] De la Calle C., Morata L., Cobos-Trigueros N., Martinez J.A., Cardozo C., Mensa J., Soriano A. (2016). Staphylococcus aureus bacteremic pneumonia. Eur. J. Clin. Microbiol. Infect. Dis..

