# Supplementary material for: Intravenous Ceftriaxone Versus Multiple Dosing Regimes of Intravenous Anti-Staphylococcal Antibiotics for Methicillin-Susceptible Staphylococcus aureus (MSSA): A Systematic Review
Source: Antibiotics (Basel). 2020 Jan 21;9(2):39. doi: 10.3390/antibiotics9020039 (PMC7169384; doi:10.3390/antibiotics9020039)
Supplement: Supplementary file 1 [file antibiotics-09-00039-s001.pdf]

Supplementary File 1. PubMed/MEDLINE (OvidSP) (1982 to July 2019)

Search (((((((ceftriaxone[Title/Abstract]) OR rocephine[Title/Abstract])) OR "Ceftriaxone"[Mesh])) AND ((((((mssa[Title/Abstract]) OR "staphylococcus aureus"[Title/Abstract]) OR "staphy A"[Title/Abstract]) OR "s aureus"[Title/Abstract])) OR (("Staphylococcus aureus"[Mesh:NoExp]) OR "Staphylococcal Infections"[Mesh])))) AND (((((((("Mortality"[Mesh]) OR (((mortality[Title/Abstract]) OR death[Title/Abstract]) OR survival[Title/Abstract])) OR "Patient Readmission"[Mesh]) OR ((readmission[Title/Abstract]) OR readmit*[Title/Abstract])) OR "Recurrence"[Mesh]) OR recur*[Title/Abstract]) OR "adverse effects" [Subheading]) OR (((toxic*) OR adverse effect*[Title/Abstract]) OR adverse event*[Title/Abstract] OR cellulitis OR Osteomyelitis OR "septic arthritis"))

Supplementary File 2. EMBASE (OvidSP) (1974 to July 2019)

ceftriaxone.mp. or ceftriaxone/
46813
2
methicillin susceptible staphylococcus aureus.mp. or methicillin susceptible Staphylococcus aureus/
4043
3
mssa.mp. [mp=title, abstract, heading word, drug trade name, original title, device manufacturer, drug manufacturer, device trade name, keyword]
3788
4
cellulitis.mp. [mp=title, abstract, heading word, drug trade name, original title, device manufacturer, drug manufacturer, device trade name, keyword]
20034
5
osteomyelitis.mp. [mp=title, abstract, heading word, drug trade name, original title, device manufacturer, drug manufacturer, device trade name, keyword]
34352
6
septic arthritis.mp. [mp=title, abstract, heading word, drug trade name, original title, device manufacturer, drug manufacturer, device trade name, keyword]
5843
7
2 or 3 or 4 or 5 or 6
62677
8
2 or 3
6001
9
1 and 8
339
10
1 and 7
2480

Supplementary File 3. Cochrane Central Register of Controlled Trials (CENTRAL 2019, Issue 7)

#1

ceftriaxone
1348
#2
MeSH descriptor: [Ceftriaxone] explode all trees
619
#3
rocephin
46
#4
#1 or #2 or #3
1350
#5
Staphylococcus aureus
2634
#6
MeSH descriptor: [Staphylococcus aureus] explode all trees
818
#7
S Aureus
1855
#8
Staphylococcal Infection*
1289
#9
1107
#10
#5 or #6 or #7 or #8 or #9
3291
#11
#4 and #10
108
#12
Adverse*
205309
#13
#11 and #12
64

Supplementary File 4. Global Health (1973 to July 2019)

Search History (17)
▼
17
3 and 12
653
Advanced
16
3 and 12 and 15
13
Advanced
15
13 or 14
34935

Advanced
14
adverse event*.ti,ab.
13363
Advanced
13
adverse effect*.ti,ab.
2225
Advanced
12
5 or 6 or 7 or 8 or 9 or 10 or 11
54668
Advanced
11
septic arthritis.ti,ab.
812
Advanced
10
osteomyelitis.ti,ab.
2478
Advanced
9
cellulitis.ti,ab.
1516
Advanced

8
Staphylococcal Infection*.ti,ab.
643
Advanced
7
S Aureus.ti,ab.
16925
Advanced
6
Staphylococcus aureus.ti,ab.
43980
Advanced
5
Staphylococcus aureus/
47488
Advanced
4
rocephin.ti,ab.
14
Advanced
3
1 or 2
5440
Advanced
2
ceftriaxone.ti,ab.

4959
Advanced
1
ceftriaxone/
4531
Advanced

Supplementary File 5. CINAHL (1996 to July 2019)

Search History
S14
S5 AND S13
Search modes—Boolean/Phrase
S13
S6 OR S7 OR S8 OR S9 OR S10 OR S11 OR S12
Search modes—Boolean/Phrase
S12
TI "staphylococcal infection*" OR AB "staphylococcal infection*"
Search modes—Boolean/Phrase
S11
TI "staphylococcus aureus" OR AB "staphylococcus aureus"
Search modes—Boolean/Phrase
S10
TI "s aureus" OR AB "s aureus"
Search modes—Boolean/Phrase
S9
TI "STAPHY A" OR AB "STAPHY A"
Search modes—Boolean/Phrase
S8
TI "Staphylococcus Aureus" OR AB "Staphylococcus Aureus"
Search modes—Boolean/Phrase
S7
(MH "Staphylococcus Aureus")
Search modes—Boolean/Phrase
S6
TI MSSA OR AB MSSA
Search modes—Boolean/Phrase
S5
S1 OR S2 OR S3 OR S4
Search modes—Boolean/Phrase
S4
TI rocephin* OR AB rocephin*
Search modes—Boolean/Phrase
S3
TI rocephine OR AB rocephine
Search modes—Boolean/Phrase
S2
TI Ceftriaxone OR AB Ceftriaxone
Search modes—Boolean/Phrase
S1
(MH "Ceftriaxone")

Search modes—Boolean/Phrase
